# Rates of self-harm presenting to general hospitals: a comparison of data from the Multicentre Study of Self-Harm in England and Hospital Episode Statistics

**DOI:** 10.1136/bmjopen-2015-009749

**Published:** 2016-02-16

**Authors:** Caroline Clements, Pauline Turnbull, Keith Hawton, Galit Geulayov, Keith Waters, Jennifer Ness, Ellen Townsend, Kazem Khundakar, Nav Kapur

**Affiliations:** 1 Centre for Mental Health and Safety, The University of Manchester, Manchester, UK; 2 Department of Psychiatry, Centre for Suicide Research, University of Oxford, Oxford, UK; 3 Derbyshire Healthcare NHS Foundation Trust, Royal Derby Hospital, Derby, UK; 4 School of Psychology, University of Nottingham, Nottingham, UK; 5 Northern and Yorkshire Knowledge and Intelligence Team, Chief Knowledge Office, Public Health England, UK; 6 Manchester Mental Health and Social Care Trust, Manchester, UK

**Keywords:** PSYCHIATRY, EPIDEMIOLOGY

## Abstract

**Objective:**

Rates of hospital presentation for self-harm in England were compared using different national and local data sources.

**Design:**

The study was descriptive and compared bespoke data collection methods for recording self-harm presentations to hospital with routinely collected hospital data.

**Setting:**

Local area data on self-harm from the 3 centres of the Multicentre Study of Self-harm in England (Oxford, Manchester and Derby) were used along with national and local routinely collected data on self-harm admissions and emergency department attendances from Hospital Episode Statistics (HES).

**Primary outcome:**

Rate ratios were calculated to compare rates of self-harm generated using different data sources nationally and locally (between 2010 and 2012) and rates of hospital presentations for self-harm were plotted over time (between 2003 and 2012), based on different data sources.

**Results:**

The total number of self-harm episodes between 2010 and 2012 was 13 547 based on Multicentre Study data, 9600 based on HES emergency department data and 8096 based on HES admission data. Nationally, routine HES data underestimated overall rates of self-harm by approximately 60% compared with rates based on Multicentre Study data (rate ratio for HES emergency department data, 0.41 (95% CI 0.35 to 0.49); rate ratio for HES admission data, 0.42 (95% CI 0.36 to 0.49)). Direct local area comparisons confirmed an overall underascertainment in the HES data, although the difference varied between centres. There was a general increase in self-harm over time according to HES data which contrasted with a fall and then a rise in the Multicentre Study data.

**Conclusions:**

There was a consistent underestimation of presentations for self-harm recorded by HES emergency department data, and fluctuations in year-on-year figures. HES admission data appeared more reliable but missed non-admitted episodes. Routinely collected data may miss important trends in self-harm and cannot be used in isolation as the basis for a robust national indicator of self-harm.

**Strengths and limitations of this study**

- The study was a novel comparison of routinely collected hospital data on self-harm and equivalent data from study centres with comprehensive and well-established data collection methods.
- The study identified the level of underascertainment for self-harm presentations within routinely collected Hospital Episode Statistics data across three study centres.
- Data on self-harm were collected from three urban areas in England making it difficult to generalise the results to less urbanised areas with different population demographics.
- Only episodes of self-harm that presented to hospitals were included rather than those taking place in the community.

## Introduction

Self-harm is recognised internationally as a common problem in health services,^1^ as well as a contributory factor to the high number of deaths by suicide worldwide.^2^ ^3^ Self-harm is one of the most common causes of hospital admission. It is often repeated,^4^ ^5^ and is associated with a range of personal and social costs.^6–8^ In England, there may be over 200 000 presentations for self-harm to emergency departments (EDs) annually,^9^ and around 20% of people who self-harm will present again to the same hospital within a year.^10^

Given the high estimates of hospital attendances due to self-harm, the impact of self-harm on the individual, and the potential severity of outcomes—particularly in relation to increased risk of repeated self-harm and suicide—it is essential that clinicians, care providers and researchers have access to accurate data. Furthermore, reliable national data on hospital presentations for self-harm could inform outcome indicators, be used to assess performance of services, and help improve overall quality of care.^11^

One way to obtain reliable data on hospital presentations for self-harm would be to use gold-standard bespoke data collection methods, such as those employed in the Multicentre Study of Self-harm in England,^10^ ^12^ which uses clinical questionnaires supplemented by searches of hospital databases. However, these methods are time consuming and resource intensive, and data are only collected from a limited number of sites. One exception to this is in the National Self-Harm Registry Ireland,^13^ but it is unclear to what extent it might be feasible to implement such registries in larger national health systems.

One alternative might be to use routinely collected hospital data which are collected nationally, then collated and published centrally. In England, Hospital Episode Statistics (HES) self-harm data from ED and inpatient settings are used in this context. These data have been quoted in health statistics and the media as overall ‘rates of self-harm’ and they have been identified as a potential source for a national indicator of self-harm.^11^ ^14^ The most reliable and established HES data relate to people who have been admitted to hospital. However, previous research has shown that only around half of people who attend hospital following self-harm are admitted to a medical bed, but there is wide variation in this proportion between hospitals.^15^ HES admission data will therefore only capture around 50% of self-harm presentations, and this proportion will vary by centre. In 2007, HES began data collection on presentations to major EDs, single specialty EDs, minor injuries units and walk-in centres in England. While ED data are not as detailed as admission data, they do include important fields such as clinical diagnoses, clinical investigations, clinical treatments and patient pathway.

The major advantage of HES data are their national scope. However, there are concerns over data coverage, clinical coding and case ascertainment.^16^ ^17^ It has been suggested that HES data do not fully capture the incidence of self-harm,^18^ but the possible extent of this has not previously been quantified. It is also unclear whether difficulties with case ascertainment in routinely collected hospital data vary by centre, or over time, or whether any particular group (eg, age group or gender) is less likely to be captured by these data sources and systematically under-represented in self-harm data.

### Aims

In this study, we aimed to compare national and local estimates of the incidence of self-harm using data collected as part of the Multicentre Study of Self-harm in England, and routinely collected hospital data available from HES. Our specific objectives were:

- To compare national rates of hospital presentation for self-harm based on Multicentre Study data, HES admission data and HES ED data.
- To compare local rates of hospital presentation for self-harm using Multicentre Study data, local HES admission data and HES ED data.
- To compare trends in hospital presentations for self-harm over time based on Multicentre Study data, and HES admission and HES ED data.

## Methods

### The Multicentre Study of Self-harm in England

Data from the Multicentre Study are collected prospectively on all individuals who present to EDs in Oxford, Manchester or Derby, following self-harm—which is defined as intentional self-poisoning or self-injury, irrespective of type of motivation including suicidal intent—regardless of whether they are admitted to a hospital bed or receive a psychosocial assessment.^19^ The study is a collaboration between the University of Oxford, the University of Manchester and Derbyshire Healthcare National Health Service (NHS) Foundation Trust. Data are collected from five general hospitals, one in Oxford, three in Manchester and one in Derby (this was previously 2 hospitals that amalgamated into 1 in 2009). Information is collected either by completion of assessment records by general hospital psychiatric service or ED staff, or by searches of ED electronic records by data collectors to identify cases not assessed by psychiatric teams. Cases are classified as self-harm via clinical codes and information generated by clinicians. An extensive list of search terms, such as ‘lacerations’, ‘psychiatric problem’ and ‘collapse’, are used to identify possible self-harm cases from electronic records, and each record is then screened for evidence that the presentation was due to self-harm. A wide range of sociodemographic and clinical information is collected, including details of the patient, method of harm, whether there was previous self-harm, psychiatric diagnosis, and if and where the patient was referred to from the ED.

Alphanumeric codes are used to uniquely identify each individual and each episode, and patient identifiable data are not shared between centres. Variables for inclusion in the core Multicentre Study data set are consistent across centres and the data set is checked for consistency and accuracy whenever new data are added. Data are relatively complete for the main sociodemographic and method of self-harm characteristics, with missing data levels between 0% and 10%. In this study, we included episodes of self-harm recorded by the Multicentre Study from January 2010 to December 2012 for the cross-sectional analysis and from January 2003 to December 2012 for analysis of trends.

We opted to use episodes rather than individuals as the basis for analyses in order to mirror the HES data most effectively, as HES data are usually presented based on episodes of patient care. Furthermore, episode-based analysis better describes the total service use of people who attend hospital after self-harm, which is important to capture given the high proportion of people who repeatedly present to the ED after self-harm. Data were restricted to residents of the local authority area covered by the Multicentre Study sites.

### Hospital Episode Statistics

HES collect data on all admissions, outpatient appointments and ED attendances at NHS hospitals in England (http://www.hscic.gov.uk/hes). Data include information on a range of clinical, demographic, administrative and geographical variables, and are available from 1989 for admitted patient care, and from 2007 for ED attendances—with 2008 being the first complete calendar year of available data. We included both admission and ED data as both have been used as sources for published rates of self-harm.

#### HES admission data

To mirror the Multicentre Study data set, we obtained^i^ HES figures on all admissions for self-harm (using the International Classification of Diseases, 10th Revision (ICD-10) codes; X60–X84, Y10–Y34 but excluding Y33.9, and Y87 for admissions), by calendar year, for local authority areas covered by the Multicentre Study. The definition of self-harm used in HES admission data is based on ICD-10 codes. The time period covered was 2010–2012 for cross-sectional analyses and 2003 to 2012 for trends. National data for the same time period, ICD-10 codes, and ‘deliberate self-harm’ patient group, were also requested for HES admission and HES ED data.

#### HES ED data

ICD-10 codes are not used to classify patients in HES ED data; instead, presentations are defined by patient group, with self-harm presentations collected under the category ‘deliberate self-harm’ (code 30). The classification of patients into the ‘deliberate self-harm’ patient group is determined by the staff undertaking the coding. Data on presentations within this patient group were included in the study. Figures were also provided split by gender and age group (≤24, 25–34, 35–54, and ≥55 years. Although self-harm is comparatively rare in those aged ≤12 years, HES data are collected for all ages and therefore we opted to include them in the analysis). HES ED data include all presentations for self-harm to EDs. This includes patients who go on to be admitted to a hospital bed (who will thereby also be recorded in HES admission data) and patients who are discharged directly from the ED. Repeat presentations by the same individual are coded as separate presentations within these data.

Although published HES ED data do include walk-in centres and minor injury units, for the purposes of this study, we were provided with only ED data (not including walk-in centres, etc) that fall under the following definition: EDs are a consultant-led 24 h service with full resuscitation facilities and designated accommodation for the reception of accident and emergency (A&E) patients.

Data were restricted to completed episodes of care for admissions within the time period (eg, any admissions that were ongoing at the end of 2012 were not included), and age groups were ascertained from age of the patients at the start of the recorded spell of treatment. All data were provided by ‘spell’ of care, which is the equivalent of a complete period of medical care from admission to discharge (HES data are sometimes presented in terms of ‘finished consultant episodes’ or FCEs, which represent a period of care under a specific consultant/specialty; a spell of care may be made up of multiple FCEs). For ease of interpretation, spells of care will hereafter be referred to as episodes or presentations, consistent with terminology commonly used in other Multicentre Study work. All HES data were anonymous and any data fields that contained less than five cases were suppressed (ie, not supplied to avoid any potential for identification of individuals).

### Ethics

All three centres have appropriate ethical and research governance approvals, are fully compliant with the Data Protection Act of 1998, and have approval under Section 251 of the NHS Act 2006 to collect patient identifiable information without patient consent.

### Analysis

A cross-section of national rates (from 2010 to 2012 inclusive, representing the most recent complete data years available) are presented, based on Multicentre Study data, HES ED data, and HES admission data. Differences in rates between data sources could be attributed to the self-harm populations in the three Multicentre Study sites being different to the self-harm population in England overall, rather than a function of case ascertainment. Therefore, we also examined a cross-section of HES ED and HES admission rates matched geographically to the local areas covered by the Multicentre Study data collection.

Rates were calculated as the number of self-harm episodes per 100 000 of the population per year, and were presented overall, and split by sex and age group. National and local authority area population estimates were taken from the Office for National Statistics (ONS)—freely available online at http://www.ons.gov.uk. Local authority area was used to calculate rates in concordance with methods commonly used in Multicentre Study research. Differences between rates based on HES ED data, HES admission data and rates based on Multicentre Study data are presented as rate ratios (RR; using Multicentre Study rates as the denominator and HES rates as the numerator) with accompanying 95% confidence intervals (CIs). As described above, in order to ensure comparability between HES and Multicentre Study data, we used an episode-based approach to analysis. This means that the absolute rates in the current analysis are likely to be higher than rates based on the number of *individuals* presenting to hospital.

National trends in hospital presentations for self-harm by men and for self-harm by women are examined based on HES emergency data and HES admission data, and compared with trends based on Multicentre Study data. Trend data covered the 10-year period of 2003–2012 as these were the years with most complete data (2008–2012 for HES ED data).

## Results

In the 3 years between 2010 and 2012, there were 329 384 presentations for self-harm to EDs in England and 332 636 admissions for self-harm, based on HES data. For the same time period, there were 13 547 self-harm episodes by people resident in the study areas presenting to hospitals in the three centres of the Multicentre Study. HES ED data covering the equivalent local area recorded 9600 self-harm presentations, and HES admission data recorded 8096 admissions to hospital beds for self-harm, between 2010 and 2012.

### National data

Cross-sectional data in table 1 show that estimated national rates of self-harm, based on HES ED and HES admission data, were substantially lower (by nearly 60%) than rates based on Multicentre Study data (RR 0.41; 95% CI 0.35 to 0.49). The differences persisted across sex and age groups, but RRs were lower for the oldest age groups for HES emergency data (RR 0.33; 95% CI 0.24 to 0.44) and for HES admission data (RR 0.40; 95% CI 0.30 to 0.53).

**Table 1:** Rates of self-harm per 100 000 per year (2010–2012), calculated using national HES admission and emergency department data, and data from the Multicentre Study of Self-harm in England

Rates (2010–2012)	Multicentre study	HES emergency department	Rate ratio* (95% CI)	HES admissions	Rate ratio* (95% CI)
Total	500.86	206.97	0.41 (0.35 to 0.49)	209.01	0.42 (0.36 to 0.49)
Men	424.47	185.58	0.44 (0.37 to 0.52)	172.83	0.41 (0.34 to 0.49)
Women	576.52	227.67	0.39 (0.34 to 0.46)	244.03	0.42 (0.36 to 0.49)
<24 years	433.31	246.90	0.57 (0.49 to 0.67)	227.99	0.53 (0.45 to 0.62)
25–34 years	662.70	332.12	0.50 (0.44 to 0.57)	314.12	0.47 (0.41 to 0.54)
35–54 years	628.46	254.73	0.41 (0.35 to 0.47)	279.59	0.44 (0.39 to 0.51)
55+ years	170.43	55.81	0.33 (0.24 to 0.44)	67.90	0.40 (0.30 to 0.53)

*Note*: All rates based on episodes not individuals.

*Rate ratios calculated using Multicentre Study data as the reference group. HES, Hospital Episode Statistics.

### Local area data

#### Manchester

Table 2 shows the rates calculated for the Manchester local area and the associated RRs. Rates based on HES ED data were much lower than rates based on the Manchester Multicentre Study data (RR 0.55; 95% CI 0.47 to 0.63). The difference between rates was fairly consistent for self-harm across subgroups, apart from in people aged 35–54 years where the RR was larger (RR 0.81; 95% CI 0.71 to 0.91), with rates per 100 000 closer to the Multicentre Study rates.

**Table 2:** Rates of self-harm per 100 000 per year (2010–2012) in Manchester

Rates (2010–2012)	Multicentre study	HES emergency department	Rate ratio* (95% CI)	HES admissions	Rate ratio* (95% CI)
Total	510.99	280.32	0.55 (0.47 to 0.63)	288.62	0.56 (0.49 to 0.65)
Men	467.62	265.95	0.57 (0.49 to 0.66)	269.64	0.58 (0.50 to 0.67)
Women	554.87	294.86	0.53 (0.46 to 0.61)	307.81	0.55 (0.48 to 0.64)
<24 years	409.49	224.60	0.55 (0.47 to 0.65)	217.77	0.53 (0.45 to 0.63)
25–34 years	684.21	344.41	0.50 (0.44 to 0.57)	346.54	0.51 (0.45 to 0.58)
35–54 years	540.58	435.62	0.81 (0.71 to 0.91)	474.80	0.88 (0.78 to 0.99)
55+ years	197.12	132.26	0.67 (0.54 to 0.84)	144.47	0.73 (0.59 to 0.91)

*Note*: all rates based on episodes not individuals.

*Rate ratios calculated using Multicentre Study data as the reference group.

HES, Hospital Episode Statistics.

Rates based on HES admission data were similar to those obtained based on HES ED data. The RR was again largest for people aged 35–54 years (RR 0.88; 95% CI 0.78 to 0.99).

#### Oxford

Table 3 shows the rates calculated for the Oxford local area. Overall, rates based on HES ED data were lower than those based on the Oxford Multicentre Study data (RR 0.66; 95% CI 0.57 to 0.77). RRs were higher for men (RR 0.85; 95% CI 0.72 to 0.1.01) and for people aged 34–54 years (RR 0.70; 95% CI 0.62 to 0.79), with HES rates closer to the Multicentre Study rates in these groups. RRs were slightly lower in women (RR 0.56; 95% CI 0.49 to 0.65) and those aged 25–34 years (RR 0.60; 95% CI 0.51 to 0.70), suggesting a bigger discrepancy between HES rates and Multicentre Study rates in these groups.

**Table 3:** Rates of self-harm per 100 000 per year (2010–2012) in Oxford

Rates (2010–2012)	Multicentre study	HES emergency department*	Rate ratio† (95% CI)	HES admissions	Rate ratio† (95% CI)
Total	423.48	275.27	0.66 (0.57 to 0.77)	290.22	0.69 (0.59 to 0.80)
Male	282.85	245.21	0.85 (0.72 to 1.01)	214.25	0.76 (0.63 to 0.90)
Female	560.94	304.97	0.56 (0.49 to 0.65)	364.31	0.65 (0.57 to 0.74)
0–24 years	438.24	278.58	0.67 (0.57 to 0.77)	260.24	0.59 (0.51 to 0.69)
25–34 years	449.06	249.55	0.60 (0.51 to 0.70)	311.33	0.69 (0.60 to 0.80)
35–54 years	587.25	425.27	0.70 (0.62 to 0.79)	465.96	0.79 (0.70 to 0.90)
55+ years	126.27	77.25	0.65 (0.48 to 0.86)	100.27	0.79 (0.61 to 1.03)

*Note:* all rates based on episodes not individuals.

*HES emergency department data restricted to 2011–2012 for Oxford due to incomplete HES emergency department data in 2010.

†Rate ratios calculated using Multicentre Study data as the reference group. For HES emergency department data, reference group was the Multicentre data for 2011–2012.

HES, Hospital Episode Statistics.

Rates based on HES admission data were similar to those obtained using HES ED data, but were lower than those based on Oxford Multicentre Study data. RRs were higher for men (RR 0.76; 95% CI 0.63 to 0.90), for those aged 34–54 years (RR 0.79; 95% CI 0.70 to 0.90) and those aged 55 years and over (RR 0.79; 95% CI 0.61 to 1.03).

#### Derby

Table 4 shows the Derby local area data. Self-harm rates based on HES ED data for Derby were very different to those calculated for the other Multicentre Study sites. Overall, the HES ED rates were around 7% *higher* than those obtained using Derby Multicentre Study data. The differences between rates changed depending on the subgroup, with RRs ranging from 0.94 (95% CI 0.75 to 1.18) for those aged 55 years and over (indicating that HES ED rates were slightly lower than Multicentre Study rates) to 1.30 (95% CI 1.15 to 1.48) for men and 1.21 (95% CI 1.10 to 1.33) for those aged 25 to 34 years (indicating that HES ED rates were higher).

**Table 4:** Rates of self-harm per 100 000 per year (2010–2012) in Derby

Rates (2010–2012)	Multicentre study	HES emergency department	Rate ratio* (95% CI)	HES admissions	Rate ratio* (95% CI)
Total	527.25	608.18	1.15 (1.03 to 1.30)	326.54	0.62 (0.54 to 0.71)
Men	422.04	550.44	1.30 (1.15 to 1.48)	256.23	0.61 (0.52 to 0.71)
Women	628.91	664.70	1.06 (0.95 to 1.18)	395.06	0.63 (0.55 to 0.71)
<24 years	486.53	501.66	1.03 (0.91 to 1.17)	312.29	0.64 (0.56 to 0.74)
25–34 years	789.46	954.10	1.21 (1.10 to 1.33)	427.46	0.54 (0.48 to 0.61)
35–54 years	802.64	848.56	1.06 (0.96 to 1.16)	504.14	0.63 (0.56 to 0.70)
55+ years	154.69	145.02	0.94 (0.75 to 1.18)	105.21	0.68 (0.53 to 0.87)

*Note*: all rates based on episodes not individuals.

*Rate ratios calculated using Multicentre Study data as the reference group.

HES, Hospital Episode Statistics.

The HES admission data for Derby were similar to the other Multicentre Study sites, with rates based on HES admission data considerably lower than the rates based on Derby Multicentre Study data (RR 0.62; 95% CI 0.54 to 0.71). This was fairly consistent across the subgroups, but the largest RR was in people aged 55 years and over (RR 0.68; 95% CI 0.53 to 0.87), indicating that the HES admission rates were closer to the Multicentre Study rates in this group.

**Figure 1:**
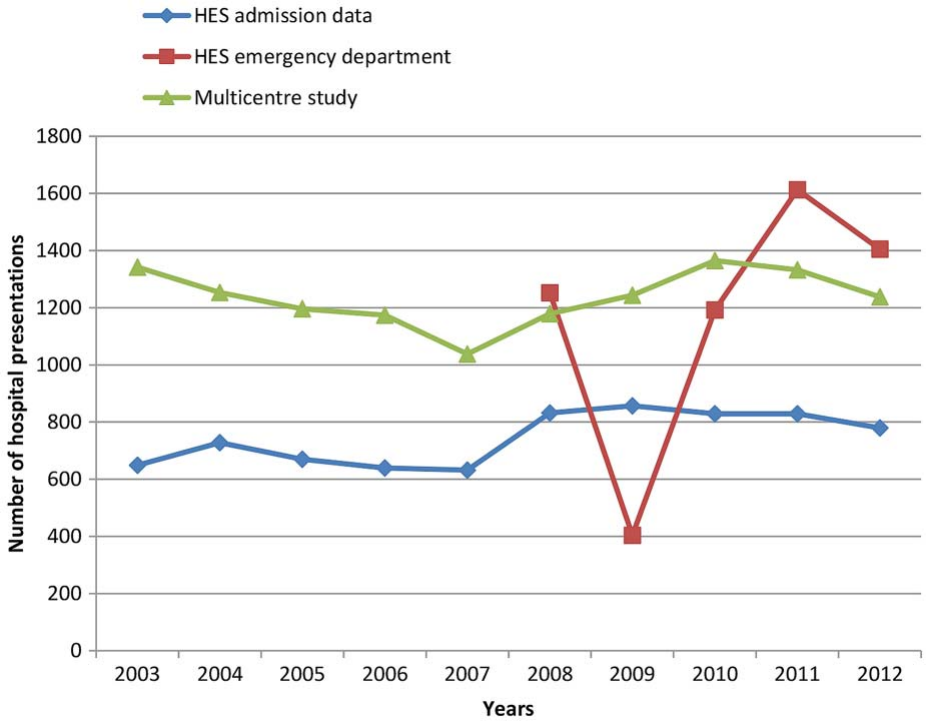
Number of hospital presentations for self-harm in Derby from 2003 to 2012 (inclusive) based on Multicentre Study data, Hospital Episode Statistics (HES) emergency department data and HES admission data.

As we were concerned about the discrepant results for HES ED data in this centre, we plotted year-on-year figures for self-harm for Derby (figure 1). Although the figures for HES admission data were lower than those from the Multicentre Study, they followed a broadly similar pattern over time. However, the number of presentations for self-harm based on HES ED data showed large year-on-year fluctuations, which may cast doubt on the robustness of these data.

### National trends

**Figure 2:**
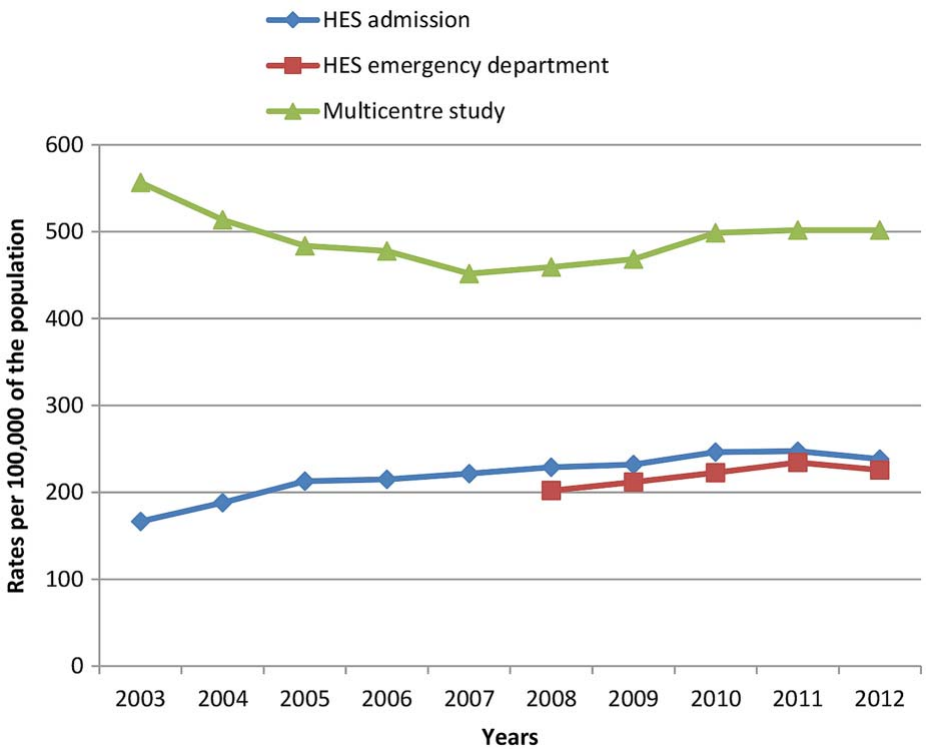
Rates of self-harm per 100 000, for all presentations by women, based on national Hospital Episode Statistics (HES) admission data, national HES emergency department data and Multicentre Study data.

**Figure 3:**
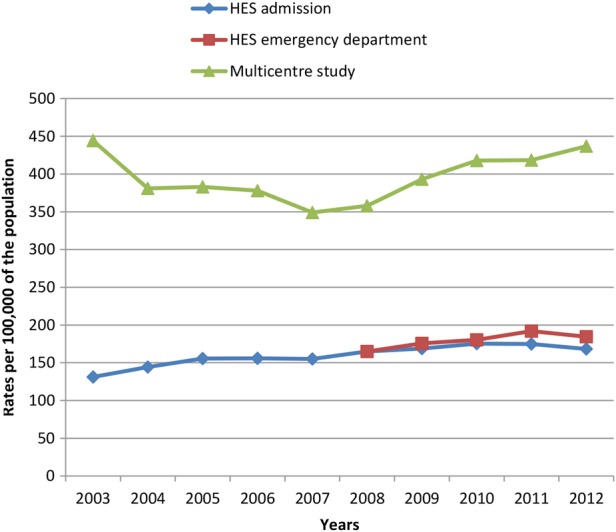
Rates of self-harm per 100 000, for all presentations by men, based on national Hospital Episode Statistics (HES) admission data, national HES emergency department data and Multicentre Study data.

Consistent with our aims, we estimated overall trends in self-harm based on national HES ED and admission data compared with trends in overall self-harm rates from the Multicentre Study (figures 2 and 3). Multicentre Study rates showed a decrease in rates of self-harm by men and women in the early part of the study period, and an increase in later years. By contrast, HES rates showed a steady increase in self-harm presentations over time, with a slight fall in 2012. The rates of self-harm by men increased markedly from 2008 in the Multicentre Study data, a trend which was not reflected to the same extent in the HES ED rates, or captured by the HES admission rates.

## Discussion

### Main findings

To our knowledge, this is the first study to directly compare routinely collected HES self-harm data with equivalent data from sites with consistent and well-established data collection methods for self-harm. We found that routine hospital data collected by HES may underestimate overall rates of self-harm by up to 60% compared with data from the Multicentre Study of Self-harm in England. The centres in this study may not be typical of the whole of England, and so we compared local area HES data to local area data collected as part of the Multicentre Study. This confirmed an overall underascertainment in the HES data although the differences varied between centres. In Manchester, rates based on HES ED data were around half of the local Multicentre Study rates, and in Oxford rates based on HES ED data were over 30% lower than Multicentre Study rates. In Derby, there were large year-on-year fluctuations in the HES ED data (eg, there was an initial decrease of 68% between 2008 and 2009, followed by a rapid increase of nearly 200% the following year) which conflicted with self-harm figures for the equivalent years in both the Multicentre Study data and the HES admission data. This could call the robustness of these data into question. One possibility is that the ‘deliberate self-harm’ category used in HES ED data collection was being used inconsistently and perhaps overapplied to a range of other mental health issues in later years. Rates based on HES admission data were consistently around 40% lower than Multicentre Study rates. Overall, self-harm rates based on both HES ED and HES admission data were closest to Multicentre Study rates for self-harm in those aged ≥55 years. Trends in self-harm rates based on the different data sources varied—Multicentre Study data showed an initial decrease over time followed by an increase (especially in men), whereas HES data showed a steady increase.

### Limitations

We used local authority areas to identify local HES data. We also restricted Multicentre Study data to those from the local authority area (based on postcode of residence) to create an equivalent data set. Broadly speaking, local authority boundaries do converge with catchment area for hospitals included in the study, although there are out of area admissions in all hospitals. However, since we used residence within local authority area as a criteria for inclusion in both HES data and Multicentre Study data, we are confident we had comparable data sets. The areas within the Multicentre Study used to calculate rates are urban and have particular population demographics. It is possible that the results are not generalisable to less urbanised areas or representative of England as a whole. The results of the local analyses, however, address this to some extent. Thus, rates of self-harm based on HES data were generally lower than self-harm rates based on the Multicentre Study, even in locations with comparatively low overall rates of self-harm. We think the same coding and data quality issues are likely to be present elsewhere, with the result that the number of hospital presentations for self-harm based on routine HES data are likely to be significant underestimates.

The automatic suppression of small values (those with a count of ≤5) within the HES data set could have artificially reduced the HES self-harm rates. However, only the local HES ED data for Oxford prior to 2011 contained suppressed values (for age groups), and as HES ED data were incomplete for earlier years, 2010 data were excluded from the local Oxford analysis. Subsequently, there were no suppressed values in the reported analysis.

Our main aim was to compare overall rates of self-harm presenting to the study centres and we included all episodes, including repeat presentations by the same person. This means that the absolute rates presented in this paper will be higher than person-based rates presented elsewhere,^10^ but this will not affect our main results which are incidence RRs comparing HES and Multicentre Study data. While individual-level analyses are important in self-harm research, an episode-based analysis represents the overall hospital-based service use due to self-harm presentations. This also allows for easier comparisons between Multicentre Study data and HES data, which are generally expressed as episodes. It is possible that our results could have been different if we had taken individuals as our unit of analysis, but this is unlikely given the size of the differences found.

This study only included hospital presentations for self-harm to compare rates based on different data sources. We did not examine the community incidence of self-harm. It is clear that a significant proportion of people who self-harm do not present to services, especially in younger age groups.^20^ However, those presenting to hospital are an important group both economically and clinically, as service use presents opportunities for intervention and prevention.

This study did not look at the differences in the capture of self-harm cases based on method of self-harm. It is possible that differences between rates from different data sources could have been greater for self-cutting than self-poisoning and this may be something to consider in any future work.

### Interpretation

Rates of self-harm based on HES data are generally lower than rates of self-harm based on data from the Multicentre Study. The difference may be a function of the method of data collection. Routinely collected hospital data are unlikely to comprehensively capture all presentations for self-harm in the same way as bespoke data collection conducted by personnel with specific expertise in identifying episodes of self-harm from hospital records. HES acknowledge that some hospitals/care providers fail to complete data submissions and that data quality may be poor in some cases (a recent update showed HES ED data captured 83% of episodes compared with NHS England's Weekly A&E reports, http://www.hscic.gov.uk/hes).

As around half of all hospital presentations for self-harm result in admission to a hospital bed, it might be expected that HES admission data would be around 50% lower than Multicentre Study and HES ED data.^15^ Previous research has shown HES admission data to be fairly accurate for recording medical diagnoses and our work shows that ICD-10 underlying cause codes for self-harm are also fairly well recorded within HES admission data.^21^ ^22^ In a post hoc analysis examining self-harm episodes resulting in inpatient admission for the years 2010–2012 inclusive, we found a 7% difference between Multicentre Study data and HES inpatient data. This varied by centre—admitted self-harm from the Multicentre Study data compared with HES admission data was 13% lower in Manchester, 8% higher in Oxford, and 6% lower in Derby. These are small differences but possible reasons for the lower incidence in two of the centres could have been related to the fact that the HES data included ICD codes for episodes of undetermined intent (some of which clinicians may not have judged to be intentional self-harm in the Multicentre Study data).

If half of patients are admitted to hospital following self-harm, rates of self-harm based on HES ED data (which includes both admitted and non-admitted episodes) should then be around twice as high as HES admission data. This was not the case, and on a national level, rates of self-harm based on HES ED data were roughly equivalent to those based on HES admission data.

HES ED data are still relatively new, being designated as experimental statistics until 2007, and continue to develop. Self-harm might be particularly difficult to code for routine statistics, as self-harm is a behaviour rather than a diagnosis and the presenting complaint may not clearly relate to the underlying cause (eg, presenting symptoms might read ‘cut’ for self-injury, or ‘stomach pain/nausea’ for overdose). Coders may not have the information or experience to extract the appropriate information needed to code correctly.^23^ ^24^ Bespoke data collection can overcome some of these difficulties but is much more labour intensive. HES recommends caution in the interpretation of the data it holds, providing quality indicators along with data extracts and citing common data quality issues including problems with data conversion, poor recording in patient notes or coding onto hospital systems, and gaps in coverage due to missed submission deadlines.^25^ ^26^

Details of how clinical coding for HES data was carried out at individual sites were beyond the scope of the current study, but it may well have varied. By contrast, Multicentre Study data collection methods are standardised and have been developed over many years. They are unlikely to have missed the number of cases indicated by HES ED data in Derby in 2011 and 2012 (with HES ED figures 21% and 13% higher, respectively), which could instead reflect differences in coding practices over time.

According to the data from the Multicentre Study, rates of self-harm presentations declined early in the study period and increased again from around 2008. This important trend—possibly related to the economic recession—could be missed if routinely collected hospital data on self-harm are relied on. Both national HES ED data and HES admission data showed a more-or-less steady increase in self-harm over time. It is possible that national trends differ from local trends, but the increase in HES admission data may be a reflection of tangible changes in care that result in more people with self-harm being admitted to a hospital bed (eg, policy restrictions on the time patients should spend in EDs and the subsequent increase in short stay beds).^15^ However, this may also reflect improvements in data collection and staff awareness of self-harm, as is likely to be the case for the HES ED data. Increasing coverage and improvements in coding could give the impression of rising numbers of self-harm presentations, and risks masking true fluctuations in the numbers of people attending hospital following self-harm—such as the recent increase in self-harm by men.

### Clinical and research implications

What is the overall burden of self-harm presenting to hospital in England? In terms of overall number of self-harm presentations to hospitals in England nationally, between 2010 and 2012 (inclusive) based on HES ED data there were on average 110 000 presentations for self-harm per year. We know this is likely to be a significant underestimate, perhaps by as much as 60%. HES admission data for the same period gives an average of approximately 111 000 admissions for self-harm per year. A recent English study suggested that 54% of self-harm episodes presenting to EDs result in hospital admission.^15^ Applying this to HES admission data might suggest the true number of self-harm episodes presenting to hospital is in the region of 205 000 annually.

Routinely collected national data on self-harm have many potential benefits but current HES data will underestimate the overall rate of self-harm. It is important that researchers, policymakers, clinicians and the media are aware of this. Recently, there has been interest in developing a national indicator for self-harm, comprising two elements: attendances at A&E for self-harm per 100 000 population, and percentage of attendances at A&E for self-harm that received a psychosocial assessment.^11^ Our findings suggest that HES data cannot be used in isolation as part of a national indicator. Routine data may also mask important trends in self-harm, for example, those related to the economic recession which follow a similar pattern to national suicide rates.^27^ ^28^ However, HES data remain potentially valuable, perhaps particularly when presented in the context of locally collected data.

HES inpatient data are robust and perhaps most useful when levels of admission of self-harm patients to medical beds are high (such as in Oxford). The quality of HES ED data are improving. The consistency of clinical coding between hospital sites, and accuracy of coding within individual sites, need to be further improved in order to increase the utility of HES ED data in relation to self-harm. Coders may need specific training on how to identify cases of self-harm from hospital records. Increased clinician involvement in coding has been proposed as a cost-effective means of improving data quality generally;^21^ ^29^ this could also help to improve national recording of self-harm. Future work might explore linking HES ED data to clinical records in order to improve case ascertainment.^30^

## Footnotes

i Data provided by the Northern and Yorkshire Knowledge and Intelligence Team, Public Health England.
